# Operando Shell‐Isolated Nanoparticle‐Enhanced Raman Spectroscopy of the NO Reduction Reaction over Rhodium‐Based Catalysts

**DOI:** 10.1002/cphc.202100375

**Published:** 2021-07-07

**Authors:** Fabiane C. Ballotin, Thomas Hartman, Joris Koek, Robin G. Geitenbeek, Bert M. Weckhuysen

**Affiliations:** ^1^ Debye Institute for Nanomaterials Science Utrecht University Universiteitsweg 99 3584 CG Utrecht The Netherlands

**Keywords:** NO reduction, operando spectroscopy, Raman Spectroscopy, rhodium, SHINERS

## Abstract

Operando shell‐isolated nanoparticle‐enhanced Raman spectroscopy (SHINERS) with on‐line mass spectrometry (MS) has been used to investigate the surface species, such as NO, NOH, NO_2_, N_2_O, and reaction products of the NO reduction reaction with CO and H_2_ over supported Rh‐based catalysts in the form of catalyst extrudates. By correlating surface intermediates and reaction products, new insights in the reaction mechanism could be obtained. Upon applying different reaction conditions (i. e., H_2_ or CO), the selectivity of the catalytic reaction could be tuned towards the formation of N_2_. Furthermore, in the absence of Rh, no reaction products were detected. The importance of the operando SHINERS as a surface‐sensitive characterization technique in the field of heterogeneous catalysis provides routes towards a better understanding of catalytic performance.

## Graphical abstract

**In the moment**: Rhodium‐based catalyst extrudates were used for operando SHINERS (shell‐isolated nanoparticle‐enhanced Raman spectroscopy) to determine simultaneously surface species such as NO, NOH, NO_2_ and products, i. e., N_2_, NH_3_, N_2_O or CO_2_ with an online Mass Spectrometry (MS). By changing the reaction conditions, the selectivity of NO reduction with H_2_ or CO could be tuned towards the desired product.

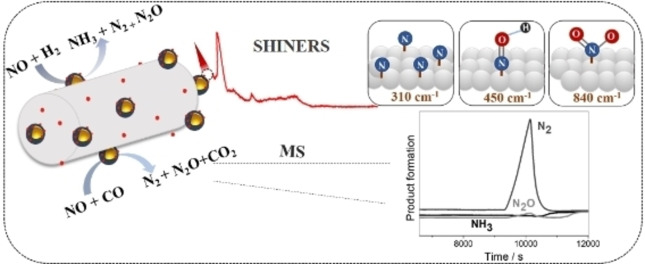

## 1 Introduction

In situ spectroscopic techniques are valuable tools to characterize catalysts and potentially surface adsorbates and intermediates during catalytically relevant conditions. This information is useful to understand and improve the physicochemical behavior of catalytic solids.[^1^, ^2^, ^3^, ^4^] However, being able to detect reaction intermediates often remains a challenge mainly due to the (very) low concentrations of these surface species.^[3]^

One analytical method that can be used for studying catalytic solids is Raman spectroscopy.^[1]^ Raman scattering, however, has a low probability, thereby making it not suitable to detect trace amounts of reactants.^[5]^ To circumvent this disadvantage, surface‐enhanced Raman spectroscopy (SERS) can be used. The technique uses the localized surface plasmon resonances on nanostructured metal surface,^[6]^ such as Au, Ag, and Cu to create strong local field enhancements.^[7]^ As a result, the Raman signal can be enhanced by factors over 10^6^. However, for practical application in catalysis, SERS substrates are required to be non‐invasive,^[8]^ and the stability of the Ag and Au nanostructures must be improved.^[9]^

To overcome these drawbacks of SERS, the method of shell‐isolated nanoparticle‐enhanced Raman spectroscopy (SHINERS) was invented.^[8]^ In SHINERS, Au or Ag nanoparticles (NPs) are coated with ultrathin layers (∼2 nm) of an inert oxide, such as SiO_2_ or TiO_2_ to obtain shell‐isolated nanoparticles (SHINs).^[8]^ The dielectric coating provides thermal and chemical stability to the plasmonic core, while preventing side‐reactions due to hot electron injection to the reactants. The SHINs can act as support material for catalytic solids, making SHINERS a powerful technique for directly probing the interaction between reactants and metals during a catalytic reaction by detecting vibrational bands and providing molecular fingerprints.[^8^, ^10^]

NOx emissions (e. g. NO, NO_2_ and N_2_O) from automotive and industrial exhaust systems, such as power generation plants and diesel trucks, are an environmental problem that has led to acid rain formation and ozone depletion.^[11]^ As a consequence, solid metal‐based catalysts have been developed^[12]^ and the ones based on Pt, Rh, and Pd^[13]^ are nowadays commonly used, being Rh the most active for NO dissociation.^[14]^ The reaction of NO and H_2_ in the presence of a metal‐based catalyst leads to the formation of NH_3_, N_2_, and N_2_O,^[15]^ while when CO is used as a reductant the reaction products are N_2_O, CO_2_ and N_2_.[^16^, ^17^]

**Figure 1:**
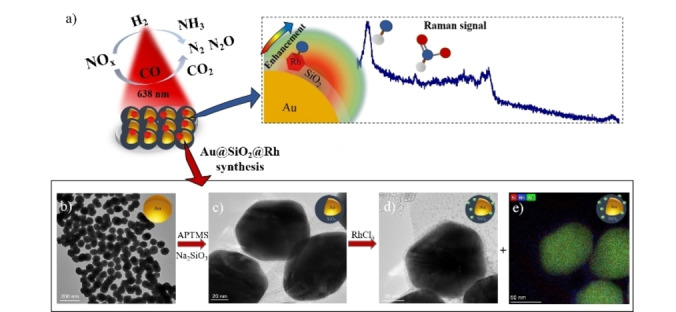
a) Schematic illustration of operando SHINERS for studying the catalytic NO_x_ reduction reaction; b) Transmission electron microscopy (TEM) image of Au nanoparticles; c) Au@SiO_2_ SHINs; d) Au@SiO_2_@Rh; e) Elemental map of Au@SiO_2_@Rh as measured with TEM, combined with energy dispersive X‐ray (EDX) analysis.

Several studies have been performed to detect the intermediates of the catalytic NO reduction using SERS[^18^, ^19^] and IR,^[20]^ but none of these investigations used SHINERS to monitor NO adsorption, desorption and reduction. In this work, we have synthesized SHINs based on Au@SiO_2_ as support materials for the preparation of Rh‐based hydrogenation catalysts (Figure 1a). The catalyst materials were used to probe NO adsorption and desorption as well as NO reduction using H_2_ and CO as reducing agent in the temperature range of 25–300 °C. Furthermore, we used catalyst extrudates to combine operando Raman spectroscopy with online mass spectrometry (MS) analysis. Using this approach, we obtained direct spectroscopic evidence of the formation of metal‐N bonds and surface intermediates as well as the formation of reaction products.

## 2 Results and Discussion

### 2.1 Preparation of SHINs

The Au nanoparticles (NPs) were synthesized using a seed‐mediated growth method and Au NPs of 78 nm were obtained (Figure 1b). According to literature, a NP size from 70–90 nm is required to obtain optimum Raman signal enhancement factors.[^10^, ^21^] The Au NPs were coated with Na_2_SiO_3_ and APTMS to form a thin SiO_2_ layer, e. g. 2.0±0.5 nm (Figure 1c), which improves the thermal stability of the plasmonic core, while minimizing the loss of Raman signal intensity and obtaining pinhole free SHINs.^[10]^ Pyridine was used as a probe molecule to demonstrate the uniformity of the SiO_2_ coating over the Au NPs and the absence of pinholes.^[22]^ We found that after coating the Au NPs with SiO_2_ there is no pyridine signal, as observed by Raman spectroscopy, showing that the Au@SiO_2_ sample made is pinhole‐free (Figure S1a). The Raman enhancement factor of the Au@SiO_2_ material was tested using the laser of 785 nm and 0.08 mW of power. 10 μL of a Rhodamine 6G solution (0.1 mM) was placed on the substrate with a cover slip to prevent drying. The dye molecule has a high Raman scattering cross‐section to probe SERS activity of plasmonic nanoparticles[^23^, ^24^] but was not in resonance with the 785 nm laser source.^[25]^ The enhancement factor was calculated according to a previous study by Le Ru *et al*.^[21]^ Based on the Raman intensity of the peak at 1362 cm^−1^ of the xanthene ring stretch of Rhodamine 6G,^[25]^ we calculated the analytical enhancement factor of the bare Au NPs and the SHINS to be approximately 5×10^5^ and 5×10^4^, respectively (Figure S1b).^[26]^

For the study of catalytic NO reduction over Rh, 10 μL of SHINs were impregnated with 10 μL RhCl_3_ (2 mM). Rh was dispersed all over the SiO_2_ layer, leading to a material further denoted as Au@SiO_2_@Rh, in which Rh particles were in the range of 0.5–1.5 nm as confirmed by TEM (Figure 1d) and Energy Dispersive X‐ray (EDX) analysis (Figure 1e).

### 2.2 NO Adsorption Experiments

**Figure 2:**
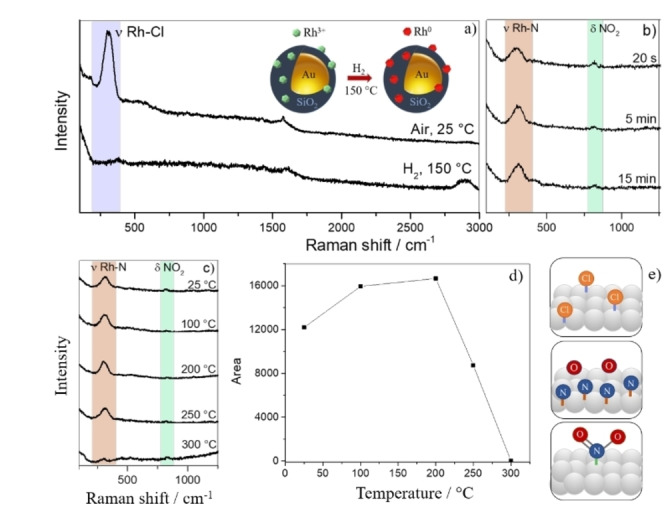
a) In situ SHINERS data of RhCl_3_ reduction (315 cm^−1^) to metallic Rh. (with λ=638 nm; P=0.22 mW); b) In situ SHINERS data for the NO adsorption onto Au@SiO_2_@Rh at 25 °C for 20 s, 5 min and 15 min; c) In situ SHINERS data for the NO adsorption on Rh with Au@SiO_2_@Rh in the temperature range of 25–300 °C (λ=638 nm; P=0.22 mW); d) Band areas of the Rh−N Raman peak for the NO adsorption experiment for Au@SiO_2_@Rh in the temperature range of 25–300 °C; e) Surface structures for adsorbed Rh−Cl, Rh−N, Rh−O, and Rh−NO_2_.

The obtained Au@SiO_2_@Rh materials were first evaluated in NO adsorption experiments. For this purpose, the samples were in situ reduced in a Linkam cell THMS600 at 150 °C for 30 min (10 mL min^−1^ H_2_ and 40 mL min^−1^ Ar), while the in situ Raman spectra were recorded using a 638 nm excitation source and a spectral resolution of 2 cm^−1^/pixel. Figure 2a shows the SHINER spectra during the reduction of RhCl_3_ to Rh^0^. Before H_2_ reduction, a Raman band at ∼310 cm^−1^, assigned to Rh−Cl stretching vibrations from the RhCl_3_ precursor, could be seen.^[27]^ Upon reducing conditions and heating, the Raman band area decreased and after 30 min at 150 °C, a featureless spectral background in the region of 310–1000 cm^−1^ could be observed, indicating the formation of metallic Rh.^[10]^

Subsequently, the Au@SiO_2_@Rh sample was cooled down to 25 °C, and NO was introduced in the in situ cell. Directly after introducing NO, a Raman band at ∼302 cm^−1^ was observed. This band can be assigned to a Rh−N stretching vibration, that originates from NO dissociation in the presence of a metal such as Rh (Figure 2b).^[19]^ Over time, the band increased in area and shifted to ∼315 cm^−1^ after 15 min of NO exposure. Next to the Rh−N vibration, a small feature was observed at ∼830 cm^−1^, which can be assigned to the bending mode of adsorbed nitro complexes (NO_2_).^[18]^ The surface NO_2_ species are formed by the reaction of NO and adsorbed oxygen.^[18]^ Typical stretching vibrations of molecular adsorbed NO, such as N−O stretching vibrations at around 2200 cm^−1[18]^ or 1700–1900 cm^−1[28]^ were not observed and Raman bands in the 1300–1600 cm^−1^ region could not be analyzed due to the presence of carbonaceous residues originating from organic carboxylic salts.[^29^, ^30^] This indicates that the catalyst synthesized in this work strongly dissociates N−O.

Figure 2c shows that the increase of temperature from 25 °C to 200 °C led to an increase in area of the Raman band at ∼315 cm^−1^ (Figure 2d), indicating an increase in the adsorption of atomic nitrogen. When the temperature reached a temperature of 300 °C, a decrease in the band area of the Raman peak at ∼315 cm^−1^ occurred, most likely related to N desorption.^[14]^ Figure 2e shows potential surface species of the type Rh−NO_2_, Rh−O, Rh−N and Rh−Cl, which can be formed after NO adsorption on a metallic Rh surface.

### 2.3 NO Reduction Experiments

**Figure 3:**
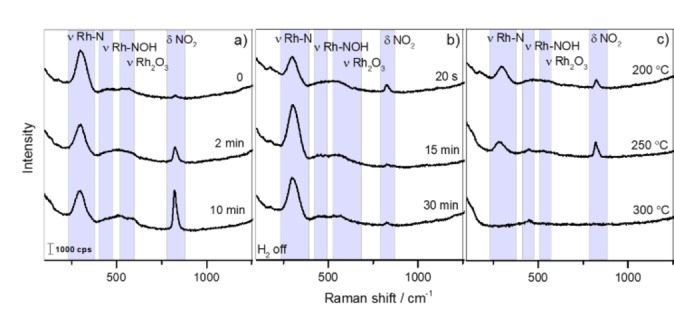
a) In situ SHINERS data of the NO reduction reaction over Au@SiO_2_@Rh, first reduced with H_2_ (H_2_ : NO ratio of 2.5 : 1) at 200 °C, for different times a) 0, 2 and 10 min; b) 20 s, 15 and 30 min in H_2_ absence; c) 200, 250 and 300 °C. (λ=638 nm; P=0.22 mW).

Figure 3a shows in situ SHINERS data for the NO reduction over the Au@SiO_2_@Rh sample with H_2_ at 200 °C. After introducing H_2_, the band area of the Rh−N stretching vibration located at ∼306 cm^−1^ decreased and shifted to ∼296 cm^−1^ after 10 min. The band shift to lower wavelengths reflects a reaction between nitrogen and hydrogen. It is also important to note that in the presence of metals such as Rh, most of the NO species dissociates, since the stretching Rh−NO at ∼260 cm^−1[31,32]^ cannot be observed in the Raman spectra. Although, Rh−N stretching vibration of an adsorbed ‐NOH species at ∼460 cm^−1^ was also detected,^[33]^ likely related to the reaction between adsorbed hydrogen and nitric oxide, a process that is favored if NO is adsorbed in a bent form,^[34]^ no OH vibration could be observed. In addition, a broad Raman band at ∼560 cm^−1^, attributed to Rh_2_O_3_,[^18^, ^33^] was present, which can be formed via a reaction between NO and metallic Rh. Although in our work Rh_2_O_3_ and NO_2_ bands were observed in H_2_ excess, Tolia and co‐workers,^[33]^ only verified these bands when an excess of NO was used. This difference might be due to the size of nanoparticles. A previous study of Rh oxidation using SERS and XPS techniques detected a Raman band at ∼505 cm^−1^, which was shifted to ∼550 cm^−1^ upon heating and was related to bulk Rh oxide.^[35]^

Moreover, the intensity of the Raman band at ∼830 cm^−1^ increased with time when H_2_ was switched on (time period of 0–15 min) (Figure 3a). In previous studies,[^33^, ^36^] this Raman band was discernible at higher temperatures, and there was no evidence of an increase of the Raman band intensity with H_2_ flow. To prove that the Raman band located at 830 cm^−1^ correlated with the presence of H_2_, the H_2_ flow was switched off (Figure 3b). Immediately, the area of the Raman band decreased, which means that H_2_ favors the NO reaction with O to subsequently form NO_2_.

Studies have shown that the NO dissociation process requires two adjacent sites and that hydrogen coverage could block it and prevent NO dissociation. As a result, the oxygen atom from NO could combine with a neighboring H atom and form a NOH surface species which was detected at 450 cm^−1[33]^ [Eq. 1].

(1) $${\;NO}_{\left(ads\right)}+\;H_{\left(ads\right)}\to {NOH}_{\left(ads\right)}+\;S\;$$

Then, the NOH surface species would react with H and OH, providing a new path for NO dissociation [Eqs. (2‐3].

(2) $${NOH}_{\left(ads\right)}+\;H_{\left(ads\right)}\to H_{2}O+N_{\left(ads\right)}+S$$

(3) $${NOH}_{\left(ads\right)}+\;{OH}_{\left(ads\right)}\to H_{2}O+{NO}_{\left(ads\right)}+S$$

To explain the increase of the NO_2_ Raman band with H_2_ flow, we propose that the adsorbed NO originating from NOH [Eq. (3)] could react with adsorbed O, produced by [Eq. (4)], thereby generating surface NO_2_ [Eq. 5]:

(4) $${NO}_{\left(ads\right)}\to {\;N}_{ads}+O_{ads}$$

(5) $${NO}_{\left(ads\right)}+\;O_{\left(ads\right)}\to {{NO}_{2}}_{\left(ads\right)}+S$$

In addition, the Raman band at ∼296 cm^−1^ shifted to ∼306 cm^−1^ and increased in intensity with time. The H_2_ was switched on again, and after 15 min, the sample was heated. In general, by increasing the temperature beyond 200 °C, the intensities of all Raman bands were affected. For example, the Raman band at ∼306 cm^−1^ shifted to ∼287 cm^−1^ and completely disappeared at 300 °C, which is in accordance with literature^[36]^ (Figure 3c).

### 2.4 NO Adsorption Experiments with Catalyst Extrudates

**Figure 4:**
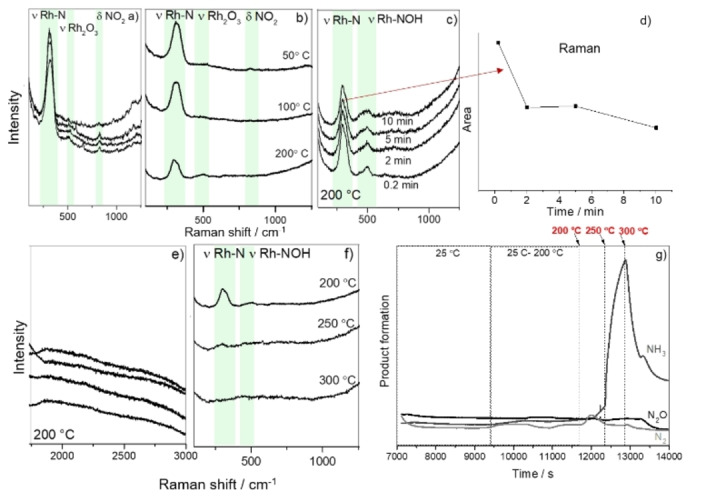
Operando SHINERS data of the NO reduction over Au@SiO_2_@Rh with H_2_ (H_2_ : NO ratio of 2.5 : 1) a) at 25 °C; b) from 50–200 °C; c, d) Area of Rh−N stretching at 200 °C; e) at 200 °C at higher energies; f) from 200–300 °C; g) Products monitored by on‐line Mass Spectrometry (MS).

**Figure 5:**
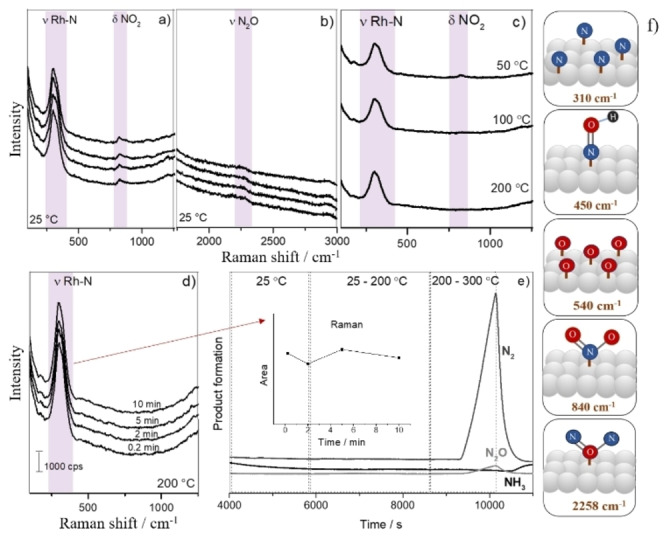
Operando SHINERS data of the NO reduction reaction over Au@SiO_2_@Rh with H_2_ (H_2_ : NO ratio of 1 : 5) a) at 25 °C, b) at 25 °C (higher energy); c) from 50–200 °C, d) at 200 °C, e) Products monitored by on‐line Mass Spectrometry (MS); inset: Band area of the Rh−N stretching band measured at 200 °C; f) Schematic illustration of the adsorbed N and NOx molecular structures on Rh surface with vibration energies shown.

**Figure 6:**
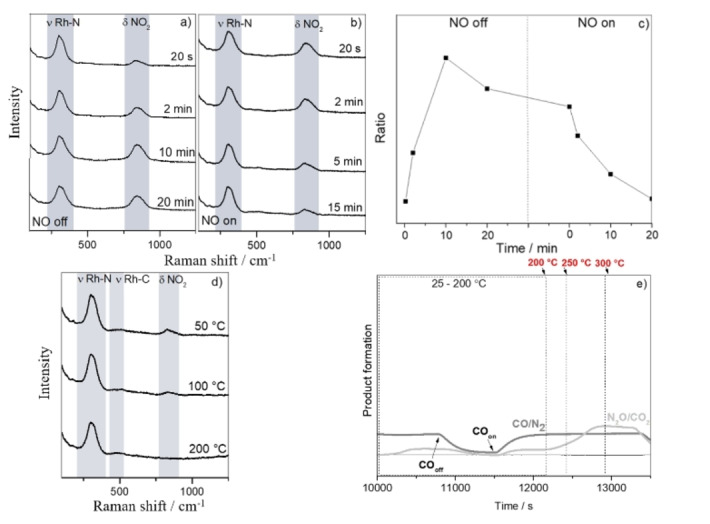
Operando SHINERS data of the NO reduction reaction over Au@SiO_2_@Rh with CO (NO : CO ratio of 2.5 : 1) at 25 °C a) after turning NO off for 20 s, 2, 10 and 20 min; b) after turning NO on for 20 s, 2, 10 and 20 min; c) Ratio of Rh−N/NO_2_−CO_2_ band area; d) from 50–200 °C; e) Products monitored by on‐line Mass Spectrometry (MS).

In another set of experiments, the Au@SiO_2_@Rh SHINs were assembled on SiO_2_ extrudates. The Au@SiO_2_ SHINs were loaded with Rh by mixing 20 μL of colloidal SHINs with 20 μL aqueous RhCl_3_ (0.1 M). The catalyst extrudate was then impregnated with the mixture and was dried under vacuum. On‐line Mass Spectrometry (MS) was used simultaneously with SHINERS to observe the catalyst activity and selectivity by following the different reaction products formed during NO reduction. The experiments were started by reduction of the catalyst at 250 °C for 30 min (10 mL min^−1^ H_2_ and 40 mL min^−1^ Ar), followed by NO reduction under four reaction conditions to understand how potential surface intermediates and reaction products change with different reducing conditions: i. e., (a) H_2_ : NO ratio of 2.5 : 1 (Figure 4); (b) H_2_ : NO ratio of 6.33 : 1 (Figure S3), (c) H_2_ : NO ratio of 1 : 5 (Figure 5); and (d) CO, CO : NO ratio of 2.5 : 1 (Figure 6).

The operando SHINERS data for the experiments conducted under a H_2_ : NO ratio of 2.5 : 1 is shown in Figure 4. Generally, the same bands observed in the previous experiment are present herein. At 25 °C, Rh−N stretching vibration, NO_2_ bending mode, and Rh−O vibration (Figure 4a) were found. As described before, when the temperature increased up to 200 °C (Figure 4b), the band area related to the Rh−N stretching peak decreased and shifted from ∼315 to ∼301 cm^−1[33]^ and the Raman band at ∼840 cm^−1^ was also attenuated. At 200 °C, the Raman spectra were recorded after 0.2, 2, 5, and 10 min (Figure 4c) and the band area of the peak at ∼315 cm^−1^ decreased with increasing time (Figure 4f). The Raman bands located at ∼530 and ∼840 cm^−1^ could no longer be observed and a new broad bands at ∼495 cm^−1^ was present (Figure 4c). The Raman band at ∼495 cm^−1^ was shifted to ∼475 cm^−1^ after 10 min and is ascribed to the Rh−NOH stretching vibration of surface NOH species.^[33]^

At 200 °C and higher frequencies (Figure 4e), no N−O stretching vibrations were detected in the Raman spectra,^[37]^ due to their low concentration, high capacity of Rh to dissociate NO in N_(ads)_+O_(ads)_, low NO dissociation energy and also to the fast reaction which occurs between N_(ads)_/O_(ads)_ and H_2_. Indeed, studies have shown that NO dissociation is highly complex and often depends on the temperature surface, surface coverage, crystal plane and concentration of surface defects.^[38]^

As observed before, the increase of the temperature from 200–300 °C led to an attenuation of all Raman bands, which were completely absent at 300 °C (Figure 4f).

Furthermore, an on‐line MS analysis was performed during the reaction, and the results are summarized in Figure 4g. At 25 °C, no reaction products were detected. The sample heating to 200 °C increased the amount of products formed, and by further increasing the time, the band area of the Rh−N Raman peak decreased (Figure 4d). From 200–300 °C, a higher catalytic activity was observed, and a large amount of NH_3_ was formed. In the operando SHINERS data, the band area of the Rh−N Raman peak vanished at 300 °C, showing that the band disappearance is highly correlated to product formation. Indeed, literature studies have shown that by increasing the temperature of NO reduction using a catalyst based on Pt/Rh up to 300 °C, NH_3_ was the main product observed, with a factor of 3 to 4 larger than N_2_ formation.^[39]^ Furthermore, a reduction study using Rh/Al_2_O_3_ showed a maximum production of NH_3_ coexisting with N_2_ at 280 °C.^[20]^

The reaction was repeated with Au@SiO_2_ SHINs without Rh catalysts (Figure S2). Immediately after flowing NO (50 mL min^−1^), a band at ∼241 cm^−1^ was present,^[32]^ which blue‐shifted to ∼265 cm^−1^ after 10 min (Figure S2a), likely related to molecular NO adsorption on Au (Au‐NO). Rhodin and co‐workers^[40]^ found that CO dissociation adsorbed on a metal surface varies with the position of substrate in the periodic table and the same conclusions were made for NO,^[38]^ which was further confirmed by Bond.^[41]^ Thus, in the case of Ag, Cu and Au metal surfaces, NO normally does not dissociate, a situation which is clearly different for a Rh metal. This occurs, because in a diatomic molecule the bond breaking occurs due to charge transfer from metal to an unoccupied antibonding molecular orbital of the adsorbate, what is favoured for metals which present low electronegativity [40], such as Rh and Ru. Pyridine tests also showed that there are no pinholes on SiO_2_ shell for Au@SiO_2_@Rh, however, for Au@SiO_2_ probably the NO was adsorbed on the Au NPs, once silica layer of the material could have pinholes smaller than pyridine, but larger than NO. In this case, NO could adsorb onto gold when Rh is not present and does not dissociates NO. At higher wavelengths, the Raman band at ∼2200 cm^−1^ (Figure S2b) was also observed and is related to N_2_O stretching mode.^[40]^

The NO reduction at 25 °C was performed and the areas of the different Raman bands were maintained. In addition, a feature at ∼446 cm^−1^, which shifted to ∼451 cm^−1^, and became narrower, and which is related to the presence of surface NOH species, was present (Figure S2c). Furthermore, by increasing the temperature up to 200 °C, the intensity of the Raman bands decreased (Figure S2d). Upon increasing the temperature up to 300 °C (Figure S2f), all Raman bands disappeared, due to NO desorption. The product formation was negligible (10^2^ less in order of magnitude) in the absence of Rh (Figure S1g), which confirms that Rh^0^ is indeed the catalyst.

The operando SHINERS data for the experiments conducted under a H_2_: NO ratio of 6.7 (Figure S3a) revealed the presence of all bands described earlier. The increase in temperature also led to a decrease in the Rh−N band intensity (Figure S3b). At 200 °C, the Raman bands at ∼530 and ∼840 cm^−1^ disappeared and the broad Raman bands at ∼485 and ∼710 cm^−1^ were also present, as observed in the above described NO reduction experiment (Figure S3c). The band area of the Rh−N peak decreased with increasing time‐on‐stream. Furthermore, no Raman bands located at higher wavelengths were also observed in the Raman spectrum (Figure S3d).

Under these conditions, all Raman bands disappeared at a reaction temperature of 250 °C (Figure S3e). Indeed, according to literature, H_2_ excess could lower the NO desorption temperature,^[33]^ confirming that H_2_ plays a role in the NO dissociation reaction. The disappearance of the Raman bands at 250 °C is slightly related to product formation as observed in the on‐line MS data. In fact, NH_3_ was the main product, and N_2_ was also formed in a large amount (Figure S3g). Studies have shown that Rh is the most active metal for NO dissociation^[14]^ and in H_2_ excess, the main product is NH_3_. The formation of NH3 is known to occur by the stepwise hydrogenation of N through the formation of surface NH_x_ species.[^42^, ^43^] On the other hand, at lower reaction temperatures, the formation of N_2_O is dominant.^[20]^

In catalysis, the reaction selectivity towards a more desirable product can usually be tuned by changing reaction conditions. Hence, we have performed a reaction with NO excess (i. e., with a H_2_: NO ratio of 1 : 5) and the corresponding operando SHINERS data are shown in Figure 5.

At 25 °C, the Rh−N Raman band was observed as well as the bending mode of NO_2_. Furthermore, the Raman band located at ∼530 cm^−1^ was not present (Figure 5a). A weak Raman band located at 2258 cm^−1^ was present in the spectrum, which can be ascribed to N_2_O adsorbed on support cations^[44]^ and which disappear upon flushing with Ar, since N_2_O weakly binds to cations[^45^, ^46^] (Figure 5b). Increasing the temperature up to 200 °C (Figure 5c) led to a decrease in the Raman band at ∼840 cm^−1^. At 200 °C (Figure 5d), the Raman band related to the NOH stretching mode was not observed, probably due to a low H_2_ concentration in the reaction cell and only the Raman band related to Rh−N was discernible.

Upon NO excess the main product was N_2_ (Figure 5e), which is different from what we observed in the previous experiments. The catalytic NO dissociation led to the formation of surface N and O atoms, and as the concentration of H_2_ is low, the N_ads_ will react either with N_ads_ or NO_ads_[^39^, ^47^] to produce N_2_,[^39^, ^48^] while NO_ads_ will react with N_ads_ to form N_2_O.^[39]^ In this case, and different from the experiments with excess of H_2_, the area of the Rh−N Raman band did not decrease in intensity at a reaction temperature of 200 °C, (Figure 5e, inset) and the main reaction products were N_2_ and N_2_O. Figure 5f and Table 1 summarizes all Raman stretching bands found in SHINERS spectra

**Table 1:** Summary of stretching and bending modes found in SHINERS spectra during NO reduction with H_2_ and CO.

Modes^[a]^	Wavelength [cm^−1^]
ν Rh‐N	∼315
ν Rh−NOH	∼450
ν Rh‐O	∼540
δ NO_2_/CO_2_	∼840
ν N_2_O	∼2258

It is known that CO is a toxic gas, which is typically found in incomplete combustion reactions^[49]^ and is responsible for NO reduction in exhaust systems, being the reduction with CO slower than with H_2_.[^50^, ^51^] In order to better understand the role of CO as reducing agent, a reaction using CO was also performed. Before introducing CO, the NO was introduced at room temperature and the Raman bands at ∼315 cm^−1^, 496 and ∼831 cm^−1^ were observed and their presence increased with time. It is important to highlight that in the presence of CO, the 831 cm^−1^ Raman band was broader than in the presence of H_2_, which is likely related to the overlap of the CO_2_ with NO_2_ band. Literature studies of CO_2_ adsorption on K‐promoted Rh surface with High‐Resolution Electron Energy Loss Spectroscopy (HREELS) showed a Raman band located at ∼820 cm^−1^ assigned to the CO_2_ bending mode.^[52]^ By switching on the CO flow, the intensity of the Raman band at ∼840 cm^−1^ also increased. No spectral features at higher wavelengths were observed.

In the presence of CO, the NO flow was switched off (Figure 6a) and the area of the Raman peak, originating from the Rh−N stretching vibration, decreased while the Raman band located at 840 cm^−1^ increased for up to 20 min of gas exposure, the peak becomes sharper and shifted position from 837 to 844 cm^−1^ which is strictly related to the presence of CO_2_. Immediately after turning the NO on again (Figure 6b), the intensity of the Raman band decreased. Figure 6c shows the ratio between the band area of the Rh−N Raman peak (located at ∼315 cm^−1^) and the band area of the NO_2_/CO_2_ surface species (located at ∼830 cm^−1^). By increasing the reaction temperature (Figure 6d), the Raman band at ∼830 cm^−1^ disappeared likely due to desorption of the surface species. Furthermore, a small band at 470 cm^−1^ was observed, which is an indication of adsorbed CO species as it can be related to Rh−CO stretching vibrations in linear position.^[10]^ When the temperature increased from 200 to 300 °C, the band Rh−N intensity decreased, which is related to NO desorption. No C−O stretching vibrations were observed at ∼2060 cm^−1^, confirming that surface N is preferentially adsorbed in comparison to CO.^[37]^

The NO reduction reaction was also monitored by an on‐line MS. In this case, production of N_2_ could not be determined, as both, N_2_ and CO species present same m/z 28. Different from what was observed when H_2_ was used as reducing agent, in this case, the reaction products were N_2_, N_2_O, and CO_2_ (Figure 6e), which is in accordance with literature results.[^17^, ^53^] Although we could not differentiate N_2_O/CO_2_, by increasing the temperature, from 200–300 °C, the signal related to N_2_O/CO_2_ increased.

When the CO flow was switched off again, we clearly observed the decreasing amounts of reaction products, namely N_2_O and N_2_, while when the CO flow was switched on the amounts of reaction products again increased.

The different reaction conditions showed a correlation between the surface intermediates and reaction products. Furthermore, when the Au@SiO_2_ was subjected to the NO reduction reaction, NO dissociation did not occur, and no reaction products were formed. In general, in the presence of excess of H_2_, NOH was the surface intermediate observed and it is believed that it is responsible for providing a new reaction pathway as showed before, that would lead to the formation of NH_3_ [Eq. (6, 7]:

(6) $$N_{\left(ads\right)}+\;H_{\left(ads\right)}\to NH+OH$$

(7) $${NH}_{\left(ads\right)}+2H_{\left(ads\right)}\to {NH}_{3}+S\;$$

On the other hand, in an excess of NO, N_ads_ will react either with N_ads_ or NO_ads_ to form N_2_ or N_2_O.

## 3 Conclusions

In this work, shell‐isolated nanoparticle‐enhanced Raman spectroscopy (SHINERS) was successfully applied to study the NO reduction over Rh. Au@SiO_2_@Rh materials were prepared to perform operando SHINERS studies of the NO reduction over supported Rh‐based catalysts with H_2_ and CO. The reactions were studied using two different SHINs substrates for in situ and operando SHINERS. By drying the SHINs with catalyst precursor on a coverslip, the materials could be used for in situ work. For operando studies, where more catalyst material is required, the SHINs and catalyst precursor were impregnated and dried on a SiO_2_ extrudate. Both methods resulted in the observation of similar Raman bands. The use of catalyst extrudates presented the advantage to simultaneously detect surface species with SHINERS up to 300 °C and to measure reactants and reaction products with online MS, making it a truly operando analytical approach. The vibrational modes of N adsorbed on Rh as well as the reaction intermediates, such as NOH, NO_2_, and the product N_2_O, were detected in situ. The presence of typical Raman Rh−N vibrational bands without the presence of RhN−O vibrations is an evidence for dissociative adsorption of NO on Rh nanostructures, and a temperature increase led to desorption and N_2_ formation. Surface NOH species were present in excess of H_2_ and are believed to be involved in an alternative reaction pathway for NO reduction, leading to NH_3_ formation. In addition, the stretching vibration, located at 2258 cm^−1^ related to that of N_2_O, as well as the broader Raman band located at 830 cm^−1^ due to CO_2_, showed that indeed reaction products can be detected using operando SHINERS. In general, from temperatures at around 200–300 °C, the Raman bands were attenuated due to surface species desorption and product formation. Under reductive conditions, the product formation was related to the presence of a NOH intermediate and the selectivity was higher for NH_3_. On the other hand, with NO excess, a selectivity toward N_2_ was observed. When CO was used as a reductant agent, N_2_ and N_2_O were the main reaction products, showing that by changing reaction conditions, the selectivity towards a more desirable product could be obtained.

## Experimental Section

### Chemicals and Gases

All chemicals were used without further purification. Sigma‐Aldrich: (3‐aminopropyl) trimethoxysilane (APTMS, 99 %), sodium silicate solution (27 %), Rhodamine 6G, RhCl_3_ (98 %), hydroxylamine hydrochloride (>98 %); Alfa Aesar: HAuCl_4_ ⋅ 3H_2_O (99.99 % metal basis); Merck: hydrochloric acid (37 %); and Acros Organics: trisodium citrate dihydrate (99 %). Water was purified with Milli‐Q system (18.2 MΩ) before use. Argon (99.999 %), hydrogen (99.999 %), oxygen (99.999 %), and nitric oxide (10 % NO in He), quality>4.5 (>99.995 %) were purchased from Linde Gas.

### SHINs Synthesis and Characterization

The detailed synthesis procedure is described in literature.^[22]^ 30 ml of mQ H_2_O and 174 μL of 1 % (w/v) HAuCl_4_ were added to a 250 mL round‐bottom flask and were brought to a boil in an oil bath, under stirring. Trisodium citrate solution (0.9 mL of 1 %(w/v) was added when the solution was boiling. The flask was removed from heat after 10 min, resulting in a ruby red colloidal solution with Au seeds. The seed dispersion (1.2 mL) was added to 112 mL of mQ water and 2 mL of 1 % (w/v) sodium silicate. The nanoparticles were grown to approximately 78 nm by adding dropwise 3.0 ml NH_2_OH×HCl (99.995 %, Sigma‐Aldrich) as a mild reducing agent and 1.71 ml of 1 % (w/v) HAuCl_4_. Colloidal Au NPs solution (15 mL) was mixed with 0.20 mL aqueous APTMS (1.0 mM) solution and stirred for 10 minutes to perform SiO_2_ growth. Subsequently, 1.6 ml of a sodium silicate solution (diluted to 0.54 % wt %, pH 10.37) was added under vigorous stirring. The flask was placed in an aluminium heating mantle at 85 °C and stirred for 50 minutes to yield Au@SiO_2_ SHINs. After synthesis, the particles were centrifuged and washed three times in mQ water and stored in 2 mL of mQ water. Subsequently, 10 μL of aqueous RhCl_3_ 2 mM solution were mixed with 10 μL of SHINs. After it, 10 μL of the mixture was dried on a cover slip in a heating plate at 40 °C. To obtain a metal‐containing catalyst, the substrate was reduced in situ by exposure to 10 mL min H_2_ in 40 mL min^−1^ Ar at 150 °C using a Linkam THMS600 heating microscope stage.

### SHINs Extrudates Synthesis and Characterization

To deposit the SHINs on catalyst extrudates, a colloidal solution of SHINs (20 μL) was mixed with 20 μL of RhCl_3_ (0.1 M). The mixture was slowly added to a dried SiO_2_ extrudate (20 mg) until the pores were filled (1 mL g^−1^). Leaving aggregated SHINs on the exterior surface of the catalyst extrudate, with a loading of 0.2 %. The obtained materials were characterized with Transmission Electron Microscopy (TEM) coupled with Energy Dispersive X‐Ray (EDX) detector. TEM‐EDX images were acquired on a FEI Talos F200X electron microscope. Samples were prepared by drop‐casting a colloidal solution on a carbon‐coated copper grid, and were left to dry in air.

### Operando SHINERS Experiments

The integration of Raman peaks was performed by applying the baseline to the spectra and measuring the area of peak at 310 cm^−1^ using the software Origin.

In order to quantify the reaction products for the catalytic NO reduction, the catalyst extrudates were reduced in situ in a Linkam cell by exposure to 10 mL min^−1^ H_2_ in 40 mL min^−1^ Ar at 250 °C using a Linkam THMS600 heating microscope stage for 20 minutes. The catalysts were submitted to NO adsorption experiments 50 mL min^−1^ NO (10 % NO in He) and further to NO reduction with H_2_ under:

- H_2_ : NO ratio of 2.5 : 1;
- H_2_ : NO ratio of 6.7 : 1;
- H_2_ : NO ratio of 1 : 5;

Furthermore, a reaction of NO reduction with CO was also performed:

- CO : NO ratio of 2.5 : 1.

The composition of the gas phase was monitored with on‐line mass spectrometry (MS) on an Omni Star GSD 320 O_2_ Analytical system (Pfeiffer Vacuum), while Raman spectroscopy measurements were performed with a Horiba Raman microscope, using a 638 nm laser excitation through×50 objective. The experiments were all performed under 1 %, with an integration time of 5 s.

### Enhancement factor

In order to show SHINERS effectiveness, the enhancement factor (EF) is usually used. Herein, the analytical enhancement factor (AEF) was used, in which we compare the Raman signal intensity of dry Rhodamine 6G with Raman signal intensity obtain from 0.1 mm of an aqueous solution of Rh6G over SHINERS substrate [Eq. 8].

(8) $$\mathrm{AEF}=\left(I_{SERS}/C_{SERS}\right)/\left(I_{RS}/C_{RS}\right)$$

In which C_SERS_=10^−7^ mol cm^−3^ and C_RS_=1.26 g cm^−3^/479.02 g mol^−1^.

For example, when we calculate the AEF of the 78 nm Au NPs with 2.0 nm SiO_2_, which has a signal intensity of 16745 counts at 1360 cm^−1^, compared with dried Rh6G with a signal intensity of 8123 counts, we obtain the enhancement factor of 5×10^4^.

## Conflict of interest

The authors declare no conflict of interest.

## Supporting information

As a service to our authors and readers, this journal provides supporting information supplied by the authors. Such materials are peer reviewed and may be re‐organized for online delivery, but are not copy‐edited or typeset. Technical support issues arising from supporting information (other than missing files) should be addressed to the authors.

Supporting Information
