# Supplementary material for: Operando Shell‐Isolated Nanoparticle‐Enhanced Raman Spectroscopy of the NO Reduction Reaction over Rhodium‐Based Catalysts
Source: Chemphyschem. 2021 Jul 7;22(15):1595–602. doi: 10.1002/cphc.202100375 (PMC8456812; doi:10.1002/cphc.202100375)
Supplement: Supplementary file 1 — Supporting Information [file CPHC-22-1595-s001.pdf]

ChemPhysChem

Supporting Information

Operando Shell-Isolated Nanoparticle-Enhanced Raman Spectroscopy of the NO Reduction Reaction over Rhodium-Based Catalysts

Fabiane C. Ballotin, Thomas Hartman, Joris Koek, Robin G. Geitenbeek, and Bert M. Weckhuysen*

Supporting Information

Supporting Information

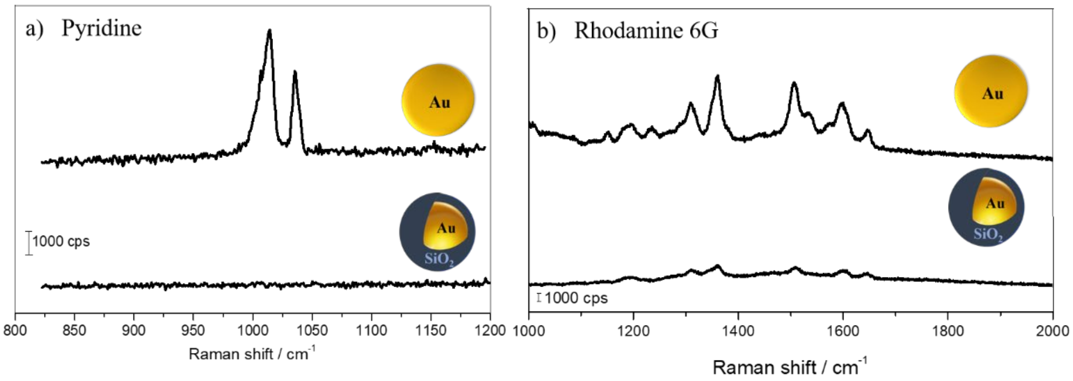

Figure S1. *In situ* Raman spectra of the enhancement and quality of the Shell-Isolated Nanoparticles (SHINs) made tested using a) pyridine as probe molecule: upon contacting the material with pyridine, cannot be observed as it is most probably not adsorbed on Au in the case of SHINS, which is distinctly different for the bare Au metal nanoparticle; b) Rhodamine 6G: upon contacting the material with Rhodamine 6G, the Raman signal intensity goes down when interacting with the SHINs relative to a bare Au metal nanoparticle.

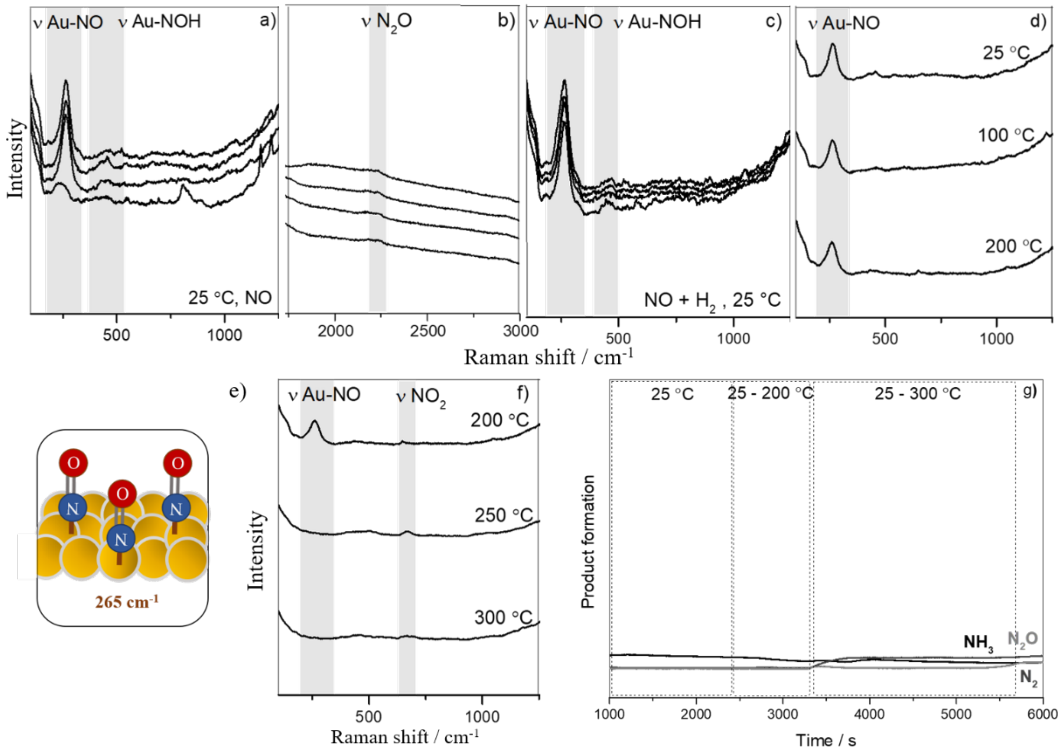

Figure S2. *In situ* Raman spectra using the synthesized Au@SiO_2 materials for a) NO adsorption at 25 °C, b) NO adsorption at 25 °C (high wavelengths) ; c) reduction with H_2 under 10 mL min^{-1} H_2 + 40.0 mL min^{-1} NO at 25 °C, d) from 25-200 °C, e) Structure of adsorbed NO on SiO_2 surface; and f) from 200-300 °C and e) on-line Mass Spectrometry (MS) data of the reaction products expected.

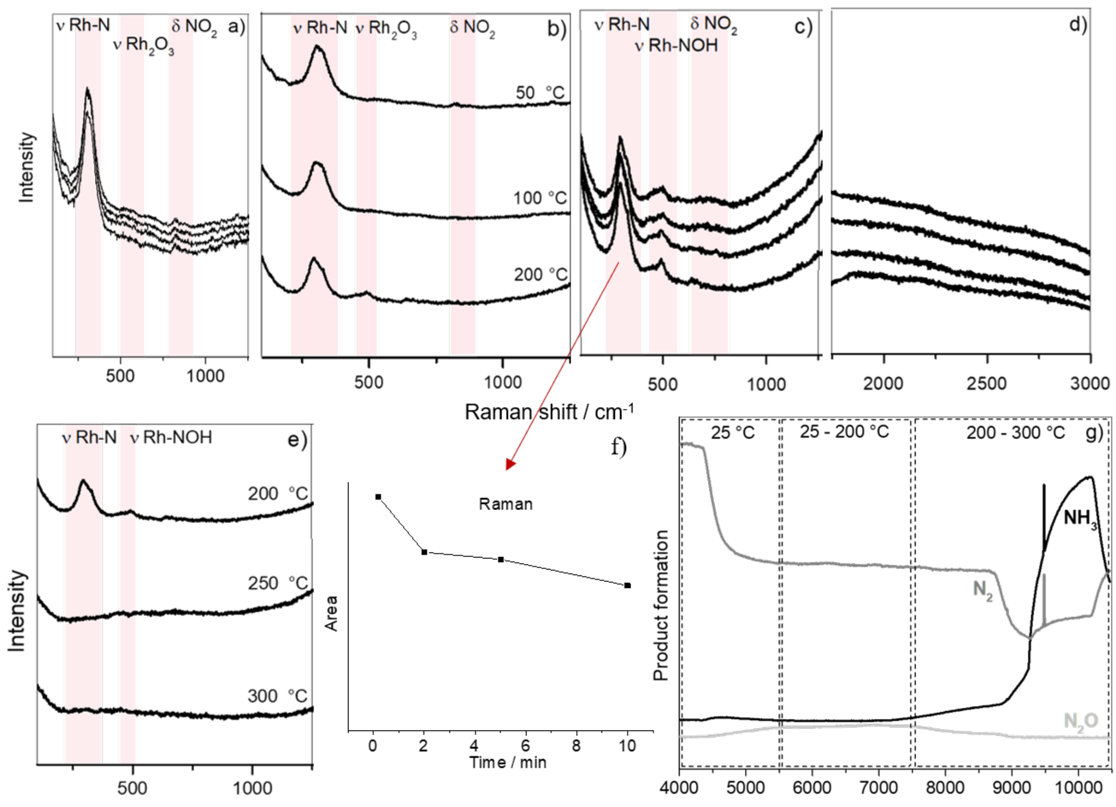

Figure S3. *Operando* Shell-Isolated Nanoparticle-Enhanced Raman Spectroscopy (SHINERS) data of the NO reduction reaction over Au@SiO_2@Rh with H_2 (H_2:NO ratio of 6.7:1) a) at 25 °C; b) from 50-200 °C; c, d) at 200 °C; e) from 200-300 °C; f) Area of the Rh-N stretching region at 200 °C; g) Evolution of the reaction products monitored by on-line Mass Spectrometry (MS).
